# Inflammatory Mechanisms of Dysmenorrhea: Novel Insights From Menstrual Effluent in an Adolescent Cohort

**DOI:** 10.1111/1471-0528.18275

**Published:** 2025-07-09

**Authors:** Chandrashekara N. Kyathanahalli, Frank F. Tu, Kevin M. Hellman

**Affiliations:** ^1^ Department of Obstetrics and Gynecology Endeavor Health Evanston Illinois USA; ^2^ Department of Obstetrics and Gynecology Pritzker School of Medicine, University of Chicago Chicago Illinois USA

**Keywords:** eicosanoids, menarche, menstruation, oxylipins, pelvic pain, prostaglandins

## Abstract

**Objective:**

To examine how eicosanoid levels in menstrual effluent of adolescents within 3 years of menarche relate to the severity of menstrual pain.

**Design:**

Prospective cohort study.

**Setting:**

Community teaching hospital.

**Population or Sample:**

Adolescents within 3 years after menarche.

**Methods:**

Participants provided a menstrual effluent sample between 4 and 30 months after menarche. Eicosanoid and oxylipin concentrations were measured in the menstrual effluent. We compared effluent concentrations of participants with menstrual pain (*n* = 33) to age‐matched pain‐free controls (*n* = 18).

**Main Outcome Measures:**

Eicosanoid and oxylipin concentrations in menstrual effluent.

**Results:**

Participants with dysmenorrhea had higher PGF2α (4.5 [1.6, 8.9] ng/mL, *p* = 0.014) than controls (1.1 [0.07, 4.4] ng/mL). However, differences in PGE2 (7.1 [2.6, 10.1] vs. 3.5 [1.0, 5.1], *p* = 0.053) and 12‐HETE (36.3 [23.7, 60.7] vs. 29.6 [13.4, 51.5], *p* = 0.305) were not significant. The correlations between PGF2α (*r* = 0.37, *p* = 0.004) or PGE2 concentration (*r* = 0.28, *p* = 0.046) and menstrual pain intensity were moderate to small. Overall, there were positive correlations between menstrual volume and eicosanoid concentrations (*r*'s > 0.4, *p*'s < 0.001). Participants with dysmenorrhea taking analgesics had more PGF2α (66.2 [43.0, 164.7]) than controls (19.1 [6.0, 47.5], *p* = 0.04). LC‐MS/MS revealed higher concentrations of 12‐HETE, 14,15‐EET, 15‐HETE, 18cdLTB4, LTB4 and PGF2α—and lower 6‐kPGF1α—in the effluent of participants with dysmenorrhea compared to controls.

**Conclusions:**

Elevated PGF2α in adolescents with dysmenorrhea, modest correlations between prostaglandin concentrations and menstrual pain, and the identification of additional oxylipins suggest that inflammatory processes beyond the prostaglandin pathway contribute to dysmenorrhea.

## 1 Introduction

Dysmenorrhea (menstrual pain) is moderate to severe in more than 40% of those of reproductive age [1, 2, 3]. Although some anatomical contributing factors (endometriosis, leiomyomas and adenomyosis) have been identified as underlying secondary dysmenorrhea, the mechanisms responsible for primary dysmenorrhea are incompletely understood [4].

It has been hypothesised that increased uterine cyclooxygenase‐2 activity increases prostaglandin synthesis, which increases uterine contractility, resulting in menstrual pain [4, 5, 6, 7]. However, this hypothesis is based on studies with less sensitive methods applied to small sample sizes of older cohorts that likely include secondary dysmenorrhea [8]. Since secondary dysmenorrhea develops later and primary dysmenorrhea emerges within a year post‐menarche, early menstrual effluent studies better reveal its underlying mechanisms [9, 10, 11]. Although NSAIDs which target COX‐2 are the first‐line treatment for menstrual pain, many adolescents with dysmenorrhea report inadequate relief, suggesting that additional inflammatory pathways may be involved [12]. Therefore, it is imperative to revisit prostaglandin's role in menstrual pain and identify other molecules objectively associated with pain severity using contemporary molecular methods in a larger sample size.

Together with prostaglandins, arachidonic acid (AA) release caused by endometrial cell breakdown during menses also produces leukotrienes (LTs, from the 5‐lipoxygenase pathway) and epoxy eicosatetraenoic acids (EETs, through the cytochrome P450 pathway), which may also influence menstrual pain and inflammatory responses [13]. For example, evidence supporting a role for the 5‐lipoxygenase pathway is a study showing that dysmenorrhea effluent has higher levels of LTC4 and LTD4 (which heighten inflammatory and smooth muscle responses) than control participants [14]. A role for the cytochrome P450 pathway is supported by a study showing that elevated 12‐hydroxyeicosatetraenoic acid (12‐HETE, which triggers inflammation by promoting leukocyte chemotaxis) was higher among participants with menstrual pain [15, 16]. Interestingly, 12‐HETE is present in concentrations an order of magnitude higher in effluent than prostaglandins, yet it has not been heavily studied to date. Therefore, evaluating uterine eicosanoid synthesis through concentration measurements in menstrual fluid could enhance our understanding of the pathogenesis of dysmenorrhea and contribute to developing new therapeutic options.

To determine whether the mechanisms identified in adults—including those with secondary dysmenorrhea—also apply to younger adolescents, we examined eicosanoid profiles in menstrual effluent. We characterised the relevant metabolites in the menstrual effluent, considering that the 5‐lipoxygenase and cytochrome P450 pathways could contribute to dysmenorrhea. Additionally, we analysed the correlation between these factors to quantify how menstrual effluent volume or NSAIDs might impact eicosanoid measurement.

## 2 Methods

### 2.1 Study Design

The current study's participants were enrolled in the Early Menstrual Pain Impact on Multisensory Hypersensitivity (EMPATHY) project, which recruited participants before menarche [17, 18]. Participants in this substudy had already completed an initial in‐person baseline assessment, which involved obtaining parental consent, adolescent assent, a general assessment of experimental pain sensitivity and comprehensive medical history questionnaires that included demographic information. They submitted a menstrual effluent sample between 4 and 30 months after menarche. The NorthShore University HealthSystem (now Endeavour Health) Institutional Review Board approved this study (EH17‐338, September 10, 2018). We waited until month 4 because period pain is unstable in the first few cycles, and studied up to month 30 because of the limited amount of time allowed by our funding [18].

### 2.2 Menstrual Symptom Diary

Participants received a written diary to submit along with the menstrual effluent sample. The diary inquired about the timing and intensity of menstrual pain on a 0–10 numerical rating scale (NRS) before inserting the pad/tampon, during the evening while using the pad/tampon, and upon removal. The prospective use of a numerical rating scale is a recognised method in pain research [19] and validated for use in studying dysmenorrhea [20]. At the end of each cycle, participants also rated their recalled level of menstrual pain and dysmenorrhea‐related impairment of school attendance, social event participation, or physical activity on a 5‐point categorical scale (never to always).

### 2.3 Collection of Menstrual Effluent

Participants were given a pre‐weighed tampon (Tampax Pearl) or pad (*always* Pure Cotton) and a precisely tared 50 mL tube. They were instructed to use the tampon or pad overnight on the first night following the onset of their menses, to remove it the following morning, and to transfer it to the tared specimen tube. Parents were asked to freeze the samples in their home freezer and return them to the lab within 48 h. Samples were kept frozen at all times or returned to the lab within 2 h of removal. On site, tampons and pads were stored at −80°C until they were needed for the assay.

### 2.4 Eicosanoid Extraction From Menstrual Effluent

The extraction protocol was modified from Hoefer et al. [15]. Pads or tampons were reweighed and soaked in 150 mL (for pads) or 30 mL (for tampons) of an acid‐alcohol mixture (0.1% formic acid in 100% HPLC/spectrophotometric ethanol; #459828 Sigma‐Aldrich, Burlington MA). They were placed on an orbital shaker set at 500 rpm for 30 min at room temperature and then extracted using a sterile syringe (for tampons) or an autoclaved stainless‐steel ricer (for pads). The extracts were centrifuged (Eppendorf, E5810R) at 2300 × *g* for 4 min at 4°C. The supernatant was saved and stored at −80°C until use.

### 2.5 Enzyme‐Linked Immunosorbent Assays

An aliquot (1.5 mL) of the alcoholic extract of menstrual effluent was evaporated to complete dryness in a Speed Vac (SC110, Savant, USA) at room temperature and then reconstituted in 10 mL of Milli‐Q water. An aliquot (100 μL) was used to measure the concentrations of Prostaglandin E2 (PGE2, ADI‐931‐001), Prostaglandin F (PGF2α, ADI‐930‐069) and 12‐HETE (ADI‐900‐050) by enzyme‐linked immunosorbent assays (ELISA), following the manufacturer's instructions (Enzo Life Sciences, Farmingdale, NY). The detection limits for these assays were 8.26, 0.98 and 146 pg/mL, respectively. The concentrations of PGE2, PGF2α and 12‐HETE were calculated using a four‐point logistic standard curve with the following characteristics: PGE2: *r* ^2^ = 0.996; PGF2α: *r* ^2^ = 0.983; and 12‐HETE: *r* ^2^ = 1.00. Each assay exhibited intra‐ and inter‐assay coefficients of variation below 10% and 15%, respectively.

### 2.6 Hemolysis Interference Measurements

Hemolysis interference (HI) was measured by the absorbance of the menstrual effluent using a SpectraMax M2 spectrophotometer (Molecular Devices, Sunnyvale, CA). A spectral scan of the alcoholic extract and water‐reconstituted menstrual effluent was performed in a 96‐well plate. Absorbance at 410 nm was recorded at the haemoglobin's peak absorption wavelength [21]. We validated this technique using blood‐extracted menstrual pads in varying cubital blood volumes. The pad's blood volume strongly correlated with absorbance at 410 nm (*r* ^2^ = 0.91).

### 2.7 Ultra‐High Performance Liquid Chromatography‐Mass Spectrometry Analysis of Eicosanoids and Oxylipins in Menstrual Effluent

We selected samples from five participants who reported the highest levels of overnight menstrual pain (NRS > 5) and five participants who reported no overnight menstrual pain for ultra‐high performance liquid chromatography‐mass spectrometry (UHPLC‐MS) analysis. The samples were submitted to Creative Proteomics (Shirley, NY) for analysis. We also submitted spiked samples to assess recovery. Additional analytical details regarding UHPLC‐MS are provided in Table S1.

### 2.8 Statistical Analysis

The original study design's sample size was determined by a power analysis to predict factors related to the development of menstrual pain [17]. For this subset of data, a post hoc sensitivity analysis using G‐power 3.1 confirmed that we had 80% power to detect differential levels of PGE2, PGF2α and 12‐HETE when comparing individuals with and without menstrual pain, with an effect size of *d* > 0.90 and *α* = 0.05, which is comparable to the existing literature (respective *d* = 0.94, 1.4, 0.92) [16, 22]. However, prior studies were not clear about the criteria used to separate participants with dysmenorrhea from controls. For instance, they did not establish cutoffs to distinguish between different levels of menstrual pain severity and participant eicosanoid levels. Therefore, we chose to compare individuals who reported no menstrual pain (rated as 0 on a 0–10 NRS) with those who indicated experiencing menstrual pain (rated as greater than 0 on a 0–10 NRS).

All statistical analyses were performed in STATA 13.1 (College Station, TX). The threshold for significance was *p* < 0.05. To estimate total content, raw concentrations of PGE2, PGF2α and 12‐HETE obtained from the ELISA were corrected for differences in extraction volume between pad (150 mL) versus tampon samples (30 mL). Thus, analyses were corrected for volume differences between collection techniques. Before statistical analysis, we also calculated the effluent concentration relative to the weight of menstrual blood collected on a tampon or pad (assuming 1 g was equal to 1 mL). Because Shapiro–Wilk tests confirmed that data were not normally distributed, nonparametric tests were used. Medians and interquartile ranges (IQRs) were calculated for the demographic variables and frequencies (percentages) for categorical variables. Group differences were evaluated using the Kruskal–Wallis test. We calculated Spearman's correlation coefficient to assess whether average menstrual pain was associated with increased pad/tampon weight or hemolysis (which could influence group differences in eicosanoid content). To verify that concentrations measured with ELISA corresponded with UHPLC‐MS measurements, we used Pearson's correlation coefficient. To analyse and interpret UHPLC‐MS data containing a large range of concentrations from multiple biomarkers, data were normalised using their *Z*‐score (mean difference/standard deviation) as recommended [23]. With two‐tailed testing, a *Z*‐score of 1.96 or greater would correspond to a significance level of *p* < 0.05.

## 3 Results

### 3.1 Demographic and Clinical Characteristics of the Study Participants

The final sample included 33 participants experiencing dysmenorrhea and 18 pain‐free controls (Figure S1). Demographic and menstrual parameters were similar between the groups except for menstrual pain intensity (Table 1, *p* < 0.001). To verify the legitimacy of the diary data, we confirmed that there was a strong correlation between diary data during the current reported menses and retrospective reports of menstrual pain at the end of the month (*r* = 0.76). Additionally, there was a high correlation between menstrual pain and the cumulative amount of missed school, social and physical activities (*r* = 0.74).

**TABLE 1:** Participants' baseline demographics and clinical characteristics of the EMPATHY cohort.

	Menstrual pain (*n* = 33)	Pain‐free (*n* = 18)	*p*
Age (years)	13.6 [13.0, 14.6]	13.7 [12.5, 14.4]	0.509
Days from menarche	265 [208, 507]	261 [223, 299]	0.65
Race			
Asian	7 (21.2%)	4 (22.2%)	1.0
Black	2 (6%)	0 (0)	0.534
Hawaiian	0 (0%)	1 (5.6%)	0.353
White	26 (78.8%)	15 (83.3%)	1.0
Hispanic	1 (3.0%)	0 (0%)	1.0
Diary symptoms			
Period pain over 24 h	**4.0 [2, 5]**	**0.0 [0, 4]**	**0.001**
Average menstrual pain during sample collection	**1.7 [0.3, 3.5]**	**0.0 [0, 0]**	**< 0.001**
Used tampon (rather than menstrual pad)	7 (21.2%)	4 (22.2%)	1.0
Length of wearing tampon or pad (hours)	11 [9, 22]	10 [9, 11]	0.075
Weight of menstrual effluent on tampon or pad (g)	7.4 [4.4, 11]	6.3 [3.1, 7.8]	0.305
Hemolytic index (abs at 410 nm)	0.07 [0.05, 0.10]	0.06 [0.05, 0.10]	0.442

*Note:* Results are reported as the median [interquartile range (IQR)] or *n* (%). Statistically significant differences are bolded. Racial percentages may not sum to 100% because some study participants were multiracial.

### 3.2 Participants With Menstrual Pain Have Higher PGF_2α_ Content in Menstrual Effluent

**FIGURE 1:**
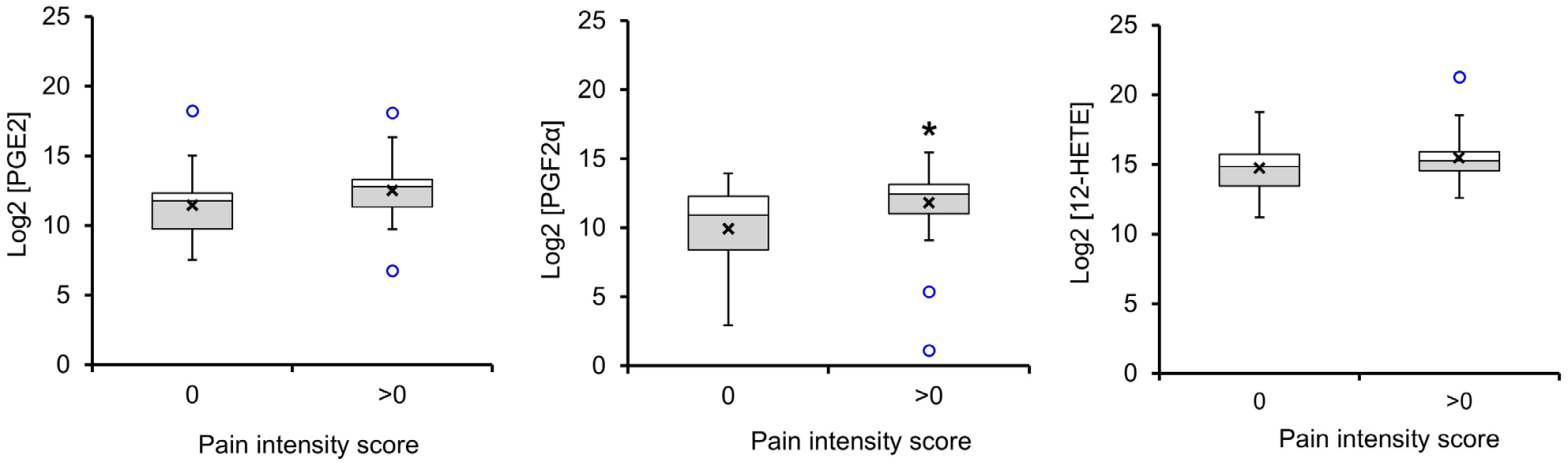
Concentrations of PGE2, PGF2α and 12‐HETE in menstrual effluent of pain‐free (NRS = 0) and menstrual pain participants (NRS ≥ 0). The concentrations were normalised to menstrual sample weight and expressed as log‐transformed data. *designates *p* < 0.05.

First, we assessed whether adolescents reporting menstrual pain had greater eicosanoid content than pain‐free controls. The median concentrations of PGF2α in menstrual effluent from those with menstrual pain (4.5 [1.6, 8.9]) were significantly higher than in controls (1.1 [0.07, 4.4]; *p* = 0.014, Figure 1 [log2 concentration values presented]). We also calculated the total amount of eicosanoids relative to the used volume of ethanol extract (Table 2). Likewise, participants with menstrual pain had higher levels of total PGF2α than pain‐free controls (Table 2, *p* = 0.04). Although median concentrations and total levels of PGE2 and 12‐HETE were 2 to 3.5 times higher in menstrual pain sufferers than in pain‐free controls, the results were not statistically significant (Table 2 and Figure 1). Notably, there was a high coefficient of variation for PGF2α (136%), PGE2 (289%) and 12‐HETE (262%) concentrations even after adjusting for group differences.

**TABLE 2:** Total amount of PGE2, PGF2α and 12‐HETE in menstrual effluent samples of pain‐free (NRS = 0) and dysmenorrhea (NRS > 0) individuals.

	Dysmenorrhea (*n* = 33)	Pain‐free (*n* = 18)	p*
PGE2 (ng/mL)	47.7 [20.1, 122.12]	22.9 [3.0, 53.6]	0.098
PGF2α (ng/mL)	31.0 [11.12, 72.4]	8.053 [0.16, 41.4]	**0.04**
12‐HETE (ng/mL)	364.3 [80.6, 647.7]	148.8 [29.2, 407.9]	0.191

*Note:* Results reported as the median [interquartile range (IQR)]. Bold and *designates *p* < 0.05.

### 3.3 Eicosanoid Concentrations Were Linked to Pain Severity and Effluent Volume/Weight

We next examined for meaningful associations between PGE2, PGF2α and 12‐HETE content, the amount of menstrual effluent collected on the tampon or pad, or the extent of hemolysis (due to more blood loss). A higher threshold for significance was used (*p* < 0.01) to account for consideration of multiple comparisons. We found the concentration of eicosanoids was positively associated with the amount of menstrual effluent collected on a tampon or pad (PGE2: *r* = 0.41, PGF2α: *r* = 0.41 and 12‐HETE: *r* = 0.58; *p*'s < 0.01). Except for PGF2α (*r* = 0.31, *p* = 0.027), the presence of hemolytic products significantly influenced detection and subsequently the menstrual effluent concentrations of PGE2 (*r* = 0.45, *p* < 0.01) and 12‐HETE (*r* = 0.67, *p* < 0.01) and the amount of blood loss and hemolysis (*r* = 0.85, *p* < 0.01). Finally, correlation coefficients were calculated to explore the associations between eicosanoid content in effluent and menstrual pain. Among the three eicosanoids measured, only the correlation between PGF2α and average menstrual pain was significant (*r* = 0.37, *p* < 0.01) (Table S2).

### 3.4 NSAID Usage Was Not Associated With Reduced Prostaglandin and 12‐HETE Content in Participants With Dysmenorrhea

We then examined whether NSAID use (which is known to inhibit prostaglandin synthesis) was associated with reduced eicosanoid content among participants with dysmenorrhea. Among the 33 dysmenorrhea participants, 11 reported the use of over‐the‐counter analgesics (such as ibuprofen, naproxen, or acetaminophen) during the use of their analysed tampon or pad (Table S3). Notably, NSAID non‐users had a significantly longer median duration of menstrual product use (tampons or pads) by approximately 4 h compared to NSAIDusers (*p* < 0.05). Thus, results could be biased in favour of finding increased eicosanoid concentrations among those who did not use NSAIDs. Because NSAIDs reduce prostaglandin levels, we expected NSAID users to have reduced prostaglandin levels. However, NSAID users unexpectedly had higher median levels of PGE2, PGF2α and 12‐HETE compared to non‐users for pain relief, but only differences for PGF2α were significant. Likewise, NSAID users had 3.3 times higher total median concentrations of PGF2α (*p* = 0.070), 1.3 times higher PGE2 (*p* = 0.445) and 1.1 times higher 12‐HETE (*p* = 0.268), but the differences were not significant.

### 3.5 Identification of Additional Eicosanoids and Oxylipins in Menstrual Effluent

To identify other molecules in menstrual effluent that might contribute to pain, we performed UHPLC‐MS on five effluent samples from adolescents experiencing the highest intensity of menstrual pain and compared them to samples from those without pain. Of the 76 eicosanoid and eicosanoid‐related metabolites assayed, 32 were reliably identified above the assay's detection limit (Table 3). Among the five participants with dysmenorrhea, we found that six of these 32 eicosanoid and related metabolites were elevated above the threshold for significance (*Z* ≥ 1.96, *p* ≤ 0.05). *Z*‐scores for significantly elevated metabolites among menstrual pain participants included 12‐HETrE (1.97), 14, 15‐EET (3.21), 15‐HETE (2.8), 18‐cdLB4 (2.24), LTB4 (3.0) and PGF2α (2.68). Conversely, 6‐kPGF1α was significantly lower in participants with dysmenorrhea compared to pain‐free controls (2.20, *p* = 0.027).

**TABLE 3:** Additional eicosanoid and eicosanoid‐related metabolites in adolescent menstrual effluent samples.

Oxylipins	Pain‐free control (*n* = 5)			Dysmenorrhea (*n* = 5)			*Z*‐score
	Mean, ng/mL	SD	% CV	Mean, ng/mL	SD	% CV	
11‐DTB2	2.243	0.718	32	1.864	0.626	34	−0.53
11‐HETE	0.194	0.122	63	0.237	0.100	42	0.36
12,13‐DiHOME	0.257	0.111	43	0.245	0.70	29	−0.11
12,13‐EpOME	0.263	0.161	61	0.451	0.304	68	1.17
12‐HETE	1.820	1.074	59	2.384	1.501	63	0.53
**12‐HETrE**	**0.824**	**0.431**	**52**	**1.675**	**1.367**	**82**	**1.97**
13,14‐dPGD2	0.181	0.052	29	0.102	0.025	24	−1.52
13‐HODE	0.272	0.204	75	0.502	0.367	73	1.13
13‐OXoODE	0.541	0.380	70	0.602	0.312	52	0.16
13‐HOTγE	0.669	0.418	62	0.681	0.361	53	0.03
**14,15‐EET**	**0.072**	**0.043**	**60**	**0.210**	**0.175**	**83**	**3.21**
**15‐HETE**	**0.69**	**0.037**	**53**	**0.173**	**0.158**	**91**	**2.80**
15‐kPGE2	0.350	0.167	48	0.303	0.277	92	−0.28
15‐LXA4	2.686	1.113	41	1.245	0.925	74	−1.30
**18‐cdLTB4**	**0.137**	**0.081**	**59**	**0.319**	**0.403**	**126**	**2.24**
5,6‐diHET	0.006	0.003	49	0.006	0.003	58	−0.25
**6‐kPGF1α**	**0.925**	**0.237**	**26**	**0.403**	**0.230**	**57**	**−2.20**
8,9‐EET	0.107	0.052	49	0.142	0.103	72	0.67
8‐HETE	0.086	0.045	53	0.118	0.068	57	0.73
9,10‐DiHOME	0.010	0.007	77	0.014	0.005	38	0.56
9,10‐EpOME	0.148	0.094	63	0.265	0.166	62	1.25
9‐HODE	0.134	0.100	75	0.233	0.164	71	0.98
9‐OXoODE	0.483	0.363	75	0.566	0.236	42	0.23
AA	3974.9	2409.7	61	5461.4	3541.8	65	0.62
**LTB4**	**0.179**	**0.053**	**29**	**0.337**	**0.425**	**126**	**3.00**
LXB4	2.528	1.380	55	0.943	0.437	46	−1.15
PGD2	2.302	0.852	37	1.558	0.280	18	−0.87
PGE2	1.042	0.394	38	0.937	0.464	50	−0.27
**PGF2α**	**1.204**	**0.309**	**26**	**2.033**	**0.940**	**46**	**2.68**
PGJ2	0.329	0.251	76	0.284	0.086	30	−0.18
ResolvinD1	2.537	2.514	99	0.907	0.586	65	−0.65
ResolvinD2	3.042	3.137	103	1.076	0.569	53	−0.63

*Note:* Dysmenorrhea participants with a *Z*‐score value of 1.97 standard deviations different than pain‐free controls (*p* < 0.05) are bolded. Results were reported as the mean (ng/mL) ± SD.

Abbreviations: 11‐DTB2, 11‐dehydrothromboxane B2; 11‐HETE, 11‐hydroxyeicosatretraenoic acid; 12,13‐DiHOME, 12,13‐dihydroxy‐9Z‐octadecenoic acid; 12,13‐EpOME, 12,13‐epoxyoctadecenoic acid; 12‐HETE, 12‐hydroxyeicosatetraenoic acid; 12‐HETrE, 12‐hydroxyeiosatrienoic acid; 13,14‐dPGD2, 13,14‐deoxyprostaglandin D2; 13‐HODE, 13‐hydroxyoctadecadienoic acid; 13‐HOTE, 13‐hydroxyoctadecatrienoic acid; 13‐OxoODE, 13‐oxo‐oxtadecadienoic acid; 14,15‐EET, 14,15‐epoxyeicosatrienoic acid; 15‐HETE, 15‐hydroxyeicosatrienoic acid; 15‐kPGE2, 15‐keto‐prostaglandin E2; 15‐LXA4, 15‐lipoxin A4; 18‐cdLTB4, 18‐carboxy dinor‐leukotriene B4; 5,6‐diHET, 5,6‐dihydroxy‐eiosatrienoic acid; 6‐kPGF1a, 6‐keto‐prostaglandin F1 alpha; 8,9‐EET, 8,9‐epoxyeicosatrienoic acid; 8‐HETE, 8‐hydroxyeicosatretraenoic acid; 9,10‐DiHOME, 9,10‐dihydroxyoctadecadienoic acid; 9,10‐EpOME, 9,10‐epoxyoctadecenoic acid; 9‐HODE, 9‐hydroxyoctadecadienoic acid; 9‐OxoODE, 9‐oxyoctadecadienoic acid; AA, arachidonic acid, LTB4, leukotriene B4; LXB4, lipoxin B4; PGD2, prostaglandin D2; PGE2, prostaglandin E2; PGF2α, prostaglandin F2 alpha; PGJ2, prostaglandin J2.

### 3.6 Correlation Analysis of Prostaglandins and 12‐HETE Concentrations Measured by ELISA and UHPLC‐MS/MS

Considering the potential impact of the extraction and analysis method on the study results and interpretation, we evaluated whether LC‐MS/MS‐based menstrual prostaglandin and 12‐HETE measures were comparable to the ELISA‐based measures. We calculated Pearson correlations for the samples analysed for PGE2, PGF2α and 12‐HETE by ELISA and UHPLC‐MS/MS methods. There was good overall agreement between LC‐MS/MS and ELISA methods in the relative concentrations for PGF2α (*r* = 0.93, *p* < 0.01) and 12‐HETE (*r* = 0.97, *p* < 0.01). However, the correlation was very weak for the PGE2 (*r* = 0.27, *p* = 0.450) concentrations reported by the two analytical methods.

## 4 Discussion

### 4.1 Main Findings

Within 3 years of menarche, adolescents reporting any degree of menstrual pain have higher concentrations of PGF2α in their menstrual effluent. The concentration of prostaglandin was correlated with effluent volume, suggesting a role in endometrial sloughing. Paradoxically, participants taking NSAIDs (which inhibit prostaglandin synthesis) had higher PGF2α concentrations than those not taking NSAIDs. Further research is needed to determine how to better inhibit PGF2α synthesis, especially at this pivotal point when menstrual pain worsens. Differences in other oxylipin metabolites (12‐HETrE, 14, 15‐EET, 15‐HETE, 18‐cdLTB4, LTB4 and 6‐kPGF1α) were also linked to menstrual pain. Future research on these molecules could uncover additional targets for treating menstrual pain.

### 4.2 Interpretation

We hypothesised that PGE2 and 12‐HETE would be elevated in adolescents with dysmenorrhea. Previous studies indicated that participants with menstrual pain had increased PGE2 in their effluent [16, 22, 24]. However, the PGE2 ELISA kit we used indicated that it was cross‐reactive to PGE1 and PGE3. Additionally, the correlations between the ELISA and UHLPC‐MS results suggest that each of our assays exhibited differences in specificity and sensitivity. Likewise, earlier studies using HPLC‐UV indicated that 12‐HETE (which is found in even higher concentrations than PGF2α) could be elevated among participants with menstrual pain [14, 16]. Although our analyses using both ELISA and UHLPC‐MS found elevated levels of 12‐HETE among participants with menstrual pain, they did not significantly differentiate pain status or correlate with pain severity. We observed a strong correlation between the ELISA and UHLPC‐MS assays, supporting the notion that both were specific and sensitive. It is possible that smaller sample sizes and the limited specificity or sensitivity of assays in prior studies contributed to this misclassification.

A UHLPC‐MS‐based targeted assay enabled us to characterise other inflammatory molecules that are differentially expressed among adolescents with menstrual pain. In our study, adolescents with more severe menstrual pain had higher concentrations of several eicosanoids and related metabolites—specifically 12‐HETrE, 14,15‐EET, 15‐HETE, 18‐cdLTB4 and LTB4—than their pain‐free counterparts. While the elevated presence of LTB4 and 15‐HETE in dysmenorrhea aligns with existing literature, the other identified metabolites represent novel findings [14, 16]. Mechanistically, these elevated metabolites could contribute to dysmenorrhea: for instance, LTB4 may promote neutrophil recruitment and exacerbate inflammation [25] and 14,15‐EET could induce uterine contractions [26]. Moreover, the observed reduction in 6‐kPGF1α levels among adolescents with menstrual pain points to a possible disruption in prostacyclin metabolism. Although 6‐kPGF1α is not biologically active, increases in its precursor prostacyclin (PGI2) could lead to heightened uterine contractility [27, 28]. Overall, these findings suggest that a combination therapy targeting COX‐2, LOX and cytochrome‐P450 pathways may provide more effective menstrual pain relief than COX‐2 inhibition alone. Potential options to consider in a future clinical trial include Zileuton (a 5‐LOX inhibitor) and GSK2256294A (a soluble epoxide hydrolase that would be expected to reduce 14,15‐EET) [29]. Although a pilot study investigated the effects of Montelukast (a leukotriene receptor inhibitor) in dysmenorrhea, it failed to demonstrate a superior effect to placebo. However, it never verified that it got a corresponding reduction in effluent inflammatory biomarkers [30]. Additionally, there was only limited use of ibuprofen to reduce COX‐2 activity in this trial. Thus, future therapeutic trials should use a combination of LOX and COX inhibitors while simultaneously evaluating the impact of drugs on menstrual effluent biomarkers.

### 4.3 Clinical Implications

Our characterisation of prostaglandins and other eicosanoid metabolites in adolescents' overnight menstrual effluent samples highlights several clinical implications. Previous studies on menstrual flow in early adolescent cohorts relied on pictorial diagrams of flow, which may introduce bias [31, 32]. In our study cohort, we previously reported at 4 months, 6.1% self‐reported using more than 6 tampons or pads on the heaviest day of their period [18]. The mass of additional effluent in our cohort was typically < 15 g (the maximum a heavy pad or tampon can reliably hold). However, 13.4% of participants had an effluent mass greater than 15 g. Thus, the need for multiple heavy tampons or pads during the night in adolescents within 3 years of menarche may be unusual but not necessarily abnormal. In our study, participants with dysmenorrhea who used NSAIDs had a 3.3 times higher content of PGF2α than those who did not take NSAIDs. Interestingly, all prior studies on the impact of NSAIDs that reported their ability to reduce PGF2α in menstrual effluent significantly used paradigms involving NSAID use starting either before or at the onset of menses, with sustained usage through day three after menses [8, 24, 33, 34, 35]. One possibility is that it is essential to use NSAIDs early (prophylactically) and also use them continuously, unlike the participants in our cohort. Future research studies should examine whether prophylactic NSAID management provides significant benefits and impacts other candidate mechanisms in menstrual pain. Also, future development of ELISA panels (which cost less than UHLPC‐MS) could be used to develop personalised treatments for dysmenorrhea by targeting the identified pathways [36, 37].

### 4.4 Strengths and Limitations

Our study is the first to utilise a combination of highly sensitive ELISAs and UHPLC‐MS to measure eicosanoid content in one of the most extensive studies of menstrual effluent to date. We also characterised effluent volume in various ways (weight, spectroscopy) to ensure that increased blood flow among participants with dysmenorrhea did not skew the measurement of eicosanoid concentrations. Validating our ability to measure from menstrual pads (instead of tampons) makes effluent studies feasible for other researchers. A key limitation is that the overnight menstrual pain was relatively low. However, our moderate correlation coefficient between PGF2α and menstrual pain indicates that even if we recruited more participants experiencing greater menstrual pain, this would not have significantly affected the findings. Due to cost, we could not apply UHPLC‐MS to all biospecimens, making it impossible to reliably correct for multiple comparisons to identify significant differences in the UHPLC‐MS analyses. Nevertheless, the UHPLC‐MS data correlated well with ELISA samples for PGF2α and 12‐HETE; thus, the findings are likely generalisable. It is possible that the PGE2 data correlated less robustly due to matrix interference related to hemolysis. However, the sample purification in ethanol and formic acid mixture should have mitigated this issue by quickly denaturing enzymes/proteins, removing the matrix and facilitating eicosanoid extraction [16]. Future studies should also evaluate genetic polymorphisms in COX‐1 and COX‐2 genes, which may also contribute to variability in inflammatory pathways, as suggested by prior studies in other gynecologic conditions [38].

## 5 Conclusions

We confirmed that PGF2α is higher in the menstrual effluent of adolescents with menstrual pain, extending findings in adults to an earlier epoch. Our adult imaging study in adults investigating the effects of naproxen also suggests dysregulation of prostaglandins plays an important role in uterine physiology and pain [39]. However, newly identified oxylipins suggest multiple inflammatory mechanisms beyond prostaglandins. The weak correlation between PGF2α and pain severity (*r* = 0.37) suggests that while prostaglandins contribute to dysmenorrhea, they are unlikely to be the sole mediators of menstrual pain. The identification of elevated leukotrienes and EETs points to a broader inflammatory network influencing pain perception. Intriguingly, NSAID users still exhibited high PGF2α, raising questions about optimal treatment strategies. Future research should explore whether dual inhibition of cyclooxygenase and lipoxygenase pathways could enhance pain relief and provide more effective dysmenorrhea treatments.

## Author Contributions

C.N.K.: conception, experimental work, editing, analysis, review, final approval. F.F.T. and K.M.H.: obtained funding, conception, editing, data interpretation, review, final approval.

## Conflicts of Interest

F.F.T. receives royalties from Wolters Kluwers and has stock options from Maipl Therapeutics. The other authors declare no conflicts of interest.

## Supporting information


**Table S1.** UHPLC fractionation gradient information.
**Table S2.** Correlation matrix examining the association of menstruation parameters and eicosanoid concentrations. * designates *p* < 0.01.
**Table S3.** PGE2, PGF2α and 12‐HETE levels in dysmenorrhea participants stratified by use. Results were reported as the median [interquartile range (IQR)].
**Figure S1.** Flowchart of the EMPATHY study population and summary of the menstrual samples from each group used for analyses in this study.

## Data Availability

The data underlying this article are available in Open Science Framework, at https://doi.org/10.17605/OSF.IO/ANCJF.

## References

[1] H. Ju , M. Jones , and G. Mishra , “The Prevalence and Risk Factors of Dysmenorrhea,” Epidemiologic Reviews 36 (2014): 104–113, 10.1093/epirev/mxt009. 24284871

[2] M. Armour , K. Parry , N. Manohar , et al., “The Prevalence and Academic Impact of Dysmenorrhea in 21,573 Young Women: A Systematic Review and Meta‐Analysis,” Journal of Women's Health 28 (2019): 1161–1171, 10.1089/jwh.2018.7615. 31170024

[3] M. E. Schoep , T. E. Nieboer , M. van der Zanden , D. D. M. Braat , and A. W. Nap , “The Impact of Menstrual Symptoms on Everyday Life: A Survey Among 42,879 Women,” American Journal of Obstetrics and Gynecology 220 (2019): 569.e1–569.e7, 10.1016/j.ajog.2019.02.048. 30885768

[4] S. Iacovides , I. Avidon , and F. C. Baker , “What We Know About Primary Dysmenorrhea Today: A Critical Review,” Human Reproduction Update 21 (2015): 762–778, 10.1093/humupd/dmv039. 26346058

[5] G. Eglinton , R. A. Raphael , G. N. Smith , W. J. Hall , and V. R. Pickles , “Isolation and Identification of Two Smooth Muscle Stimulants From Menstrual Fluid,” Nature 200 (1963): 960, 10.1038/200960a0. 14097744

[6] J. A. Maybin , H. O. D. Critchley , and H. N. Jabbour , “Inflammatory Pathways in Endometrial Disorders,” Molecular and Cellular Endocrinology 335 (2011): 42–51, 10.1016/j.mce.2010.08.006. 20723578

[7] K. M. Hellman , C. S. Kuhn , F. F. Tu , et al., “Cine MRI During Spontaneous Cramps in Women With Menstrual Pain,” American Journal of Obstetrics and Gynecology 218 (2018): 506.e1–506.e8, 10.1016/j.ajog.2018.01.035. PMC5916049 29409786

[8] W. Y. Chan , M. Y. Dawood , and F. Fuchs , “Relief of Dysmenorrhea With the Prostaglandin Synthetase Inhibitor Ibuprofen: Effect on Prostaglandin Levels in Menstrual Fluid,” American Journal of Obstetrics and Gynecology 135 (1979): 102–108. 474640

[9] M. S. Arruda , “Time Elapsed From Onset of Symptoms to Diagnosis of Endometriosis in a Cohort Study of Brazilian Women,” Human Reproduction 18 (2003): 756–759, 10.1093/humrep/deg136. 12660267

[10] O. Yu , D. Scholes , R. Schulze‐Rath , J. Grafton , K. Hansen , and S. D. Reed , “A US Population‐Based Study of Uterine Fibroid Diagnosis Incidence, Trends, and Prevalence: 2005 Through 2014,” American Journal of Obstetrics and Gynecology 219 (2018): 591.e1–591.e8, 10.1016/j.ajog.2018.09.039. 30291840

[11] O. Widholm , “Dysmenorrhea During Adolescence,” Acta Obstetricia et Gynecologica Scandinavica. Supplement 87 (1979): 61–66, 10.3109/00016347909157792. 288295

[12] P. R. Owen , “Prostaglandin Synthetase Inhibitors in the Treatment of Primary Dysmenorrhea. Outcome Trials Reviewed,” American Journal of Obstetrics and Gynecology 148 (1984): 96–103, 10.1016/s0002-9378(84)80039-3. 6419611

[13] J. Kikut , N. Komorniak , M. Ziętek , J. Palma , and M. Szczuko , “Inflammation With the Participation of Arachidonic (AA) and Linoleic Acid (LA) Derivatives (HETEs and HODEs) is Necessary in the Course of a Normal Reproductive Cycle and Pregnancy,” Journal of Reproductive Immunology 141 (2020): 103177, 10.1016/j.jri.2020.103177. 32659532

[14] S. Nigam , C. Benedetto , M. Zonca , et al., “Increased Concentrations of Eicosanoids and Platelet‐Activating Factor in Menstrual Blood From Women With Primary Dysmenorrhea,” Eicosanoids 4 (1991): 137–141. 1772686

[15] G. Hofer , C. Bieglmayer , B. Kopp , and H. Janisch , “Measurement of Eicosanoids in Menstrual Fluid by the Combined Use of High Pressure Chromatography and Radioimmunoassay,” Prostaglandins 45 (1993): 413–426, 10.1016/0090-6980(93)90118-q. 8321911

[16] C. Bieglmayer , G. Hofer , C. Kainz , A. Reinthaller , B. Kopp , and H. Janisch , “Concentrations of Various Arachidonic Acid Metabolites in Menstrual Fluid Are Associated With Menstrual Pain and Are Influenced by Hormonal Contraceptives,” Gynecological Endocrinology 9 (1995): 307–312, 10.3109/09513599509160464. 8629459

[17] F. F. Tu , K. M. Hellman , G. E. Roth , K. E. Dillane , and L. S. Walker , “Noninvasive Bladder Testing of Adolescent Females to Assess Visceral Hypersensitivity,” Pain 163 (2022): 100–109, 10.1097/j.pain.0000000000002311. 34086630 PMC8505577

[18] F. F. Tu , K. M. Hellman , S. E. Darnell , et al., “A Multidimensional Appraisal of Early Menstrual Pain Experience,” American Journal of Obstetrics and Gynecology 230 (2024): 550.e1–550.e10, 10.1016/j.ajog.2024.01.017. PMC11165568 38290643

[19] M. A. Ferreira‐Valente , J. L. Pais‐Ribeiro , and M. P. Jensen , “Validity of Four Pain Intensity Rating Scales,” Pain 152 (2011): 2399–2404, 10.1016/j.pain.2011.07.005. 21856077

[20] G. T. De Arruda , P. Driusso , J. C. Rodrigues , A. G. de Godoy , and M. A. Avila , “Numerical Rating Scale for Dysmenorrhea‐Related Pain: A Clinimetric Study,” Gynecological Endocrinology 38 (2022): 661–665, 10.1080/09513590.2022.2099831. 35850576

[21] A. Ishiguro , M. Nishioka , A. Morishige , et al., “Determination of the Optimal Wavelength of the Hemolysis Index Measurement,” Journal of Clinical Medicine 12 (2023): 5864, 10.3390/jcm12185864. 37762805 PMC10531830

[22] M. A. Lumsden , R. W. Kelly , and D. T. Baird , “Primary Dysmenorrhoea: The Importance of Both Prostaglandins E_2_ and F_2α_ ,” BJOG: An International Journal of Obstetrics and Gynaecology 90 (1983): 1135–1140, 10.1111/j.1471-0528.1983.tb06460.x. 6580910

[23] S. R. Khan , Y. Manialawy , M. B. Wheeler , and B. J. Cox , “Unbiased Data Analytic Strategies to Improve Biomarker Discovery in Precision Medicine,” Drug Discovery Today 24 (2019): 1735–1748, 10.1016/j.drudis.2019.05.018. 31158511

[24] A. M. Powell , W. Y. Chan , P. Alvin , and I. F. Litt , “Menstrual‐PGF2 Alpha, PGE2 and TXA2 in Normal and Dysmenorrheic Women and Their Temporal Relationship to Dysmenorrhea,” Prostaglandins 29 (1985): 273–290, 10.1016/0090-6980(85)90208-4. 3856904

[25] P. V. Afonso , M. Janka‐Junttila , Y. J. Lee , et al., “LTB4 Is a Signal‐Relay Molecule During Neutrophil Chemotaxis,” Developmental Cell 22 (2012): 1079–1091, 10.1016/j.devcel.2012.02.003. 22542839 PMC4141281

[26] E. Gonzalez , A. Jawerbaum , V. Novaro , and M. A. Gimeno , “Influence of Epoxyeicosatrienoic Acids on Uterine Function,” Prostaglandins, Leukotrienes, and Essential Fatty Acids 56 (1997): 57–61, 10.1016/s0952-3278(97)90525-1. 9044437

[27] J. Van Dam , T. M. Fitzpatrick , L. S. Friedman , et al., “Cardiovascular Responses to 6‐Keto‐Prostaglandin E1 in the Dogs,” Proceedings of the Society for Experimental Biology and Medicine 166 (1981): 76–79, 10.3181/00379727-166-41027. 7008057

[28] B. Jana , J. J. Jaroszewski , J. Czarzasta , M. Włodarczyk , and W. Markiewicz , “Synthesis of Prostacyclin and Its Effect on the Contractile Activity of the Inflamed Porcine Uterus,” Theriogenology 79 (2013): 470–485, 10.1016/j.theriogenology.2012.10.020. 23218395

[29] C.‐P. Sun , X.‐Y. Zhang , C. Morisseau , et al., “Discovery of Soluble Epoxide Hydrolase Inhibitors From Chemical Synthesis and Natural Products,” Journal of Medicinal Chemistry 64 (2021): 184–215, 10.1021/acs.jmedchem.0c01507. 33369424 PMC7942193

[30] Z. Harel , S. Riggs , R. Vaz , P. Flanagan , and D. Harel , “The Use of the Leukotriene Receptor Antagonist Montelukast (Singulair) in the Management of Dysmenorrhea in Adolescents,” Journal of Pediatric and Adolescent Gynecology 17 (2004): 183–186, 10.1016/j.jpag.2004.03.037. 15125904

[31] J. Sanchez , S. Andrabi , J. L. Bercaw , and J. E. Dietrich , “Quantifying the PBAC in a Pediatric and Adolescent Gynecology Population,” Pediatric Hematology and Oncology 29 (2012): 479–484, 10.3109/08880018.2012.699165. 22866673

[32] J. L. Magnay , S. O'Brien , C. Gerlinger , et al., “Pictorial Methods to Assess Heavy Menstrual Bleeding in Research and Clinical Practice: A Systematic Literature Review,” BMC Women's Health 20 (2020): 24, 10.1186/s12905-020-0887-y. 32041594 PMC7011238

[33] M. O. Pulkkinen and A. I. Csapo , “Effect of Ibuprofen on Menstrual Blood Prostaglandin Levels in Dysmenorrheic Women,” Prostaglandins 18 (1979): 137–142, 10.1016/s0090-6980(79)80031-3. 392621

[34] M. Y. Dawood and F. S. Khan‐Dawood , “Differential Suppression of Menstrual Fluid Prostaglandin F2a, Prostaglandin E2, 6‐Keto Prostaglandin F1a and Thromboxane B2 by Suprofen in Women With Primary Dysmenorrhea,” Prostaglandins & Other Lipid Mediators 83 (2007): 146–153, 10.1016/j.prostaglandins.2006.10.009. 17259081

[35] M. Y. Dawood and F. S. Khan‐Dawood , “Clinical Efficacy and Differential Inhibition of Menstrual Fluid Prostaglandin F2alpha in a Randomized, Double‐Blind, Crossover Treatment With Placebo, Acetaminophen, and Ibuprofen in Primary Dysmenorrhea,” American Journal of Obstetrics and Gynecology 196 (2007): 35.e1–35.e5, 10.1016/j.ajog.2006.06.091. 17240224

[36] X. Yu , N. Schneiderhan‐Marra , and T. O. Joos , “Protein Microarrays for Personalized Medicine,” Clinical Chemistry 56 (2010): 376–387, 10.1373/clinchem.2009.137158. 20075183 PMC7108201

[37] A. Nayyar , M. I. Saleem , M. Yilmaz , et al., “Menstrual Effluent Provides a Novel Diagnostic Window on the Pathogenesis of Endometriosis,” Frontiers in Reproductive Health 2 (2020): 3, 10.3389/frph.2020.00003. 36304708 PMC9580670

[38] S. Pandey , R. D. Mittal , M. Srivastava , K. Srivastava , and B. Mittal , “Cyclooxygenase‐2 Gene Polymorphisms and Risk of Cervical Cancer in a North Indian Population,” International Journal of Gynecological Cancer 20 (2010): 625–630, 10.1111/IGC.0b013e3181c63f79. 20686383

[39] R. H. Cockrum , F. F. Tu , O. Kierzkowska , N. Leloudas , P. V. Pottumarthi , and K. M. Hellman , “Ultrasound and Magnetic Resonance Imaging‐Based Investigation of the Role of Perfusion and Oxygen Availability in Menstrual Pain,” American Journal of Obstetrics and Gynecology 230 (2024): 553.e1–553.e14, 10.1016/j.ajog.2024.01.018. PMC11070298 38295969

