# Supplementary material for: Inflammatory Mechanisms of Dysmenorrhea: Novel Insights From Menstrual Effluent in an Adolescent Cohort
Source: BJOG. 2025 Jul 9;132(11):1626–34. doi: 10.1111/1471-0528.18275 (PMC12258968; doi:10.1111/1471-0528.18275)
Supplement: Supplementary file 1 — Table S1. UHPLC fractionation gradient information. Table S2. Correlation matrix examining the association of menstruation parameters and eicosanoid concentrations. * designates p < 0.01. Table S3. PGE2, PGF2α and 12‐HETE levels in dysmenorrhea participants stratified by use. Results were reported as the median [interquartile range (IQR)]. Figure S1. Flowchart of the EMPATHY study population and summary of the menstrual samples from each group used for analyses in this study. [file BJO-132-1626-s001.docx]

**Supplementary Material, Table, and Figures**

*UHPLC-MS/MS analysis:* Analysis of eicosanoids and their metabolites in menstrual effluent was performed at Creative Proteomics (Shirley, NY). Briefly, 1 mL of alcoholic extract of menstrual effluent from pain-free (NRS=0, n=5) and dysmenorrhea (NRS >5, n=5) mixed with an isotopically-labeled internal standard mixture containing 76 oxylipins was lyophilized, reconstituted in 300 µL methanol and sonicated for 180s at 65Hz. After adding ethyl acetate (1 mL), the samples were centrifuged (12000 rpm, 10 min, 4°C), and the supernatant was evaporated to dryness under a stream of nitrogen. The residue was reconstituted in 150 µL methanol. The clear supernatant was subjected to UHPLC-MS/MS analysis after centrifugation (12000 rpm, 10 min, 4°C).

The UHPLC separation was performed using an AB SCIEX 5500 LC system equipped with a Waters Acquity UPLC BEH C18 column (100 mm X 2.1 mm, 1.7 µm). The mobile phases A and B were 0.1% formic acid in water and 0.1 % formic acid in acetonitrile, respectively. The temperature of the column was set to 40°C. The temperature of the sample chamber was set to 4°C, with a flow rate of 0.3 mL/min for 10 min. The injection volume of all samples was 10 µL.

For assay development, a SCIEX 6500 QTRAP+triple quadrapole mass spectrometer (ScieX), equipped with an Ion Drive Turbo V electrospray ionization (ESI) interface was used. Typical ion source parameters were as follows: Temperature: 450°C; Ion Spray Voltage: -4500V; curtain gas: 35 psi; Ion Source Gas 1: 55 psi; and Ion Source Gas 2:55 psi. The Q1/Q3 pairs with the highest sensitivity and selectivity were used for quantitative monitoring.

There was 30% recovery with a spiked PGF2α sample, 120% recovery for PGE2 (potential matrix interference), and 6% recovery for 12-HETE.

Considering the potential impact of the extraction and analysis method on the study results and interpretation, we evaluated whether LC-MS/MS-based menstrual prostaglandin and 12-HETE measures were comparable to the ELISA-based measures. We calculated Pearson correlations for the samples analyzed for PGE2, PGF2α, and 12-HETE by ELISA and UHPLC-MS/MS methods. There was good overall agreement between LC-MS/MS and ELISA methods in the relative concentrations for PGF2α (r =0.93, p<0.01) and 12-HETE (r=0.97, p<0.01). However, the correlation was very weak for the PGE2 (r=0.27, p=0.450) concentrations reported by the two analytical methods.

**Supplementary Table S1**: UHPLC fractionation gradient information

| **Time (min)** | **A%** | **B%** |
| --- | --- | --- |
| 0 | 95 | 5 |
| 1 | 95 | 5 |
| 1.5 | 25 | 75 |
| 8.6 | 10 | 90 |
| 9.0 | 95 | 5 |
| 10.0 | 95 | 5 |

**Supplementary Table S2.** Correlation matrix examining the association of menstruation parameters and eicosanoid concentrations. * designates p < 0.01

|  | PGE2 | PGF2α | HETE-12 | Effluent weight | Hemolytic Index |
| --- | --- | --- | --- | --- | --- |
| PGF2α | **0.54*** |  |  |  |  |
| HETE-12 | **0.44*** | **0.40*** |  |  |  |
| Effluent weight | **0.41*** | **0.41*** | **0.58*** |  |  |
| Hemolytic Index | **0.45*** | 0.31 | **0.67*** | **0.85*** |  |
| Average menstrual pain | 0.28 | **0.37*** | 0.17 | 0.12 | 0.16 |

**Supplemental Table S3.**

**Table 3**: PGE2, PGF2α, and 12-HETE levels in dysmenorrhea participants stratified by use.

|  | No NSAIDs | NSAID-Use | p-value^*^ |
| --- | --- | --- | --- |
| *n* | 22 | 11 |  |
| Length of wearing tampon or pad (hours) | 13.5 [10,22] | 9.3 [8.9,11.6] | **0.045** |
| Menstrual pain over 24 hours | 3.0 [2.0 5.0] | 4.0 [3.0,6.5] | 0.537 |
| Average menstrual pain during sample collection | 1.6 [0.3,3.4] | 2.0 [0.5,4.6] | 0.270 |
| Hemolytic index (410 nm) | 0.06 [0.05,0.09] | 0.08 [0.06, 0.18] | 0.097 |
| Total amount of PGE2 (ng/mL) | 40.2 [18.6, 93.7] | 71.4 [25.0,186.2] | 0.311 |
| Total amount of PGF2α (ng/mL) | 19.1 [6.0,47.5] | 66.2 [43.0,164.7] | **0.04** |
| Total amount of 12-HETE (ng/mL) | 199.0 [64.0,632.7] | 426.4 [206.1,1242.0] | 0.36 |
| PGE2 (ng/g menstrual effluent) | 6.9 [3.0, 8.9] | 8.8 [1.9,24.5] | 0.44 |
| PGF2α (ng/g menstrual effluent) | 2.5 [1.1, 6.5] | 8.6 [5.0,15.4] | 0.07 |
| 12-HETE (ng/g menstrual effluent) | 36.0 [19.4, 54.9] | 38.9 [26.7,253.6] | 0.27 |

Results were reported as the median [interquartile range (IQR)].

**Supplemental Figure S1**

**Total enrolled**

N=375

**Total qualified**

n**=** 51

**Excluded from studies:**

- 1 Invalid collection
- 1 Improper storage
- 322 Participants were unable or not interested

**Pain-free controls**

NRS: 0/10 (n=18)

**Menstrual Pain**

NRS: >0/10 (n=33)

**Dysmenorrhea**

(n=5)

**Dysmenorrhea**

(n=33)

**Pain-free controls (n=5)**

**Menstrual effluent (n=51)**

**ELISA: PGE2, PGF2α, 12-HETE**

**UHPLC-MS/MS: Eicosanoids and eicosanoid-related metabolites profile**

**Pain-free controls (n=18)**

**Used NSAID**

(n=11)

**No NSAID (n=22)**

Figure 1

**Figure 1: Flowchart of the EMPATHY study population and summary of the menstrual samples from each group used for analyses in this study.**
